# Beyond COVID-19 Infection: Cognitive and Emotional Pathways Between Posttraumatic Stress, Rumination, and Quality of Life in Hospitalized Patients

**DOI:** 10.3390/healthcare13141655

**Published:** 2025-07-09

**Authors:** Margarida Vilaça, Sandra Carvalho, Jorge Leite, Fernanda Leite, M. Graça Pereira

**Affiliations:** 1 Research Centre in Psychology (CIPsi), School of Psychology, University of Minho, 4710-057 Braga, Portugal; margaridavilaca@psi.uminho.pt; 2 Psychological Neuroscience Laboratory, Research Centre in Psychology (CIPsi), School of Psychology, University of Minho, 4710-057 Braga, Portugal; sandrarc@psi.uminho.pt; 3 Basic Psychology Department, School of Psychology, University of Minho, 4710-057 Braga, Portugal; 4 RISE-Health, CINTESIS.UPT, Portucalense University, 4200-072 Porto, Portugal; jorgel@upt.pt; 5 Institute for Research and Innovation in Health (i3S), University of Porto, 4200-135 Porto, Portugal; fernandajtleite@hotmail.com; 6 Department of Transfusion Medicine, Santo António University Hospital Center, 4050-342 Porto, Portugal; 7 Public Health and Forensic Sciences, and Medical Education Department, Faculty of Medicine, University of Porto, 4200-450 Porto, Portugal; 8 Applied Psychology Department, School of Psychology, University of Minho, 4710-057 Braga, Portugal

**Keywords:** COVID-19 patients, quality of life, posttraumatic stress, rumination, cognitive functioning, emotional functioning, loneliness, posttraumatic growth

## Abstract

**Background**: Hospitalization during the COVID-19 pandemic has been linked with increased psychological distress, cognitive impairment, and reduced quality of life (QoL). Posttraumatic stress symptoms (PTSS) and rumination may significantly influence QoL outcomes, yet the mechanisms underlying these effects remain poorly understood. Based on the Cognitive Aging Model, this study examines the mediating effects of cognitive and emotional functioning, loneliness, and posttraumatic growth (PTG) on the connection between PTSS/rumination and QoL among patients hospitalized with COVID-19, including the moderator effect of sex, time since discharge, and admission to the intensive care unit (ICU). **Methods**: A cohort of 258 patients previously hospitalized with COVID-19 as the primary or secondary diagnosis was assessed 6 to 24 months post-discharge. Participants completed validated self-report and neuropsychological assessments of PTSS, rumination, cognitive function, psychological morbidity (depression and anxiety), loneliness, PTG, and QoL. Path analysis and multigroup analysis were employed to assess mediating and moderating effects. **Results**: PTSS and rumination were associated with reduced physical and mental QoL, primarily via increased psychological morbidity, impaired cognitive functioning, loneliness, and reduced PTG. Rumination showed strong direct and indirect effects on multiple mediators. Only sex and time since discharge significantly moderated pathways, with women showing a strong association between rumination and cognitive impairment/loneliness, while the association between loneliness and mental QoL was significant only in men and in recently discharged patients. **Conclusions**: PTSS and rumination contribute negatively to QoL in post-discharged patients with COVID-19 through emotional, cognitive, and social pathways, influenced by sex and duration since discharge. The findings underscore the significance of comprehensive long-term care methods focused on cognitive rehabilitation, psychosocial sT, and social reintegration for COVID-19 survivors.

## 1. Introduction

The rapid spread of SARS-CoV-2 worldwide led the World Health Organization to declare the outbreak in January 2020 and a pandemic on11 March2020, causing an unprecedented health, economic, and social crisis [1]. In Portugal, the first COVID-19 patients were confirmed on 2 March 2020, and since then, more than 5,500,000 infections and 28,000 associated deaths have been reported [2]. As expected, there was an increase in hospitalizations due to COVID-19, particularly during the winter months and the peaks of various waves. Portugal’s highest number of COVID-19 hospitalizations occurred in February 2021, with over 6800 hospitalized patients, including 904 in intensive care units (ICUs) [3,4].

The COVID-19 pandemic represented a serious threat to physical and mental health, resulting in a global collective traumatic experience, often expressed through posttraumatic stress symptoms (PTSS) [5,6]. The experience of COVID-19, whether through direct exposure to the infection or indirect exposure via media coverage, may have contributed to the incidence of posttraumatic stress disorder (PTSD) [7]. Previous studies have shown the relationship between the COVID-19 pandemic and increased levels of PTSS [7,8], with 4.6% to 55.3% of the general population reporting PTSS related to the pandemic [9]. Additionally, a study conducted by COVID-19 Mental Disorders Collaborators [10] found an increase of 28% and 26% in the prevalence of major depression and anxiety disorders, respectively, due to the COVID-19 pandemic during 2020.

Particularly, hospitalized patients infected with SARS-CoV-2 reported significant mental health challenges, with approximately 13.2% suffering from PTSD, 21% from depression, and 16.4% from anxiety [11]. Notably, the prevalence of these conditions was higher among females [9,11,12] and ICU patients [13,14]. As a result, severe COVID-19 patients reported reduced quality of life (QoL) [15] with no improvements during the first 12 months post-discharge [16].

The experience of life stressors, such as hospitalization due to COVID-19, may contribute to ruminative thoughts, i.e., repetitive and intrusive thoughts focused on negative experiences and emotions, which have been associated with depression and anxiety symptoms [17,18], being a significant predictor of PTSS in COVID-19 patients [17]. Previous studies have also demonstrated the adverse effect COVID-19-related rumination has on QoL [19].

Individuals with PTSS may also experience posttraumatic growth (PTG), which refers to positive psychological changes that can occur following a life crisis or traumatic event [20]. Several studies have shown positive associations between PTG and the experience of COVID-19 [21], particularly among discharged COVID-19 female patients (e.g., [22]), suggesting that these patients can process and elaborate the pandemic experience positively, contributing to greater satisfaction with life and QoL [23,24].

During the pandemic, physical distancing measures were required to control the virus spread, increasing loneliness and contributing to worse QoL [25,26]. Feelings of loneliness are particularly prevalent among older patients hospitalized due to COVID-19 [27,28], which is concerning given that loneliness is a well-established risk factor for increased morbidity and mortality [29].

Due to the inflammatory process in COVID-19 infection, cognitive impairment has been extensively reported in the literature, indicating a compromise in executive functions, attention, and memory [30], particularly in women [31]. Patients who required hospitalization tend to exhibit more pronounced cognitive decline than those treated in outpatient settings, presenting mild to severe cognitive impairment [32,33]. Additionally, cognitive symptoms may significantly impact daily functioning, leading to emotional distress and reduced QoL [34,35].

To advance understanding of the psychological and cognitive consequences of COVID-19 hospitalization, this study examined the contribution of trauma-related variables, namely PTSS and rumination, to QoL, considering potential pathways through cognitive performance and impairment, psychological morbidity (depression and anxiety), loneliness, and PTG. These interrelations remain insufficiently explored in the current literature, particularly across diverse cohorts of COVID-19 patients. Furthermore, hypothesized moderators such as sex, time since hospital discharge, and disease severity (e.g., ICU admission) warrant further investigation about their impact on QoL. Addressing these gaps is essential for informing the development of more targeted and effective interventions to support the long-term well-being of COVID-19 patients.

Based on the previous literature, we hypothesize that impaired cognitive functioning, increased psychological morbidity, greater loneliness, and less PTG will mediate the negative relationship between PTSS/rumination and QoL (Hypothesis 1). We further expect these associations to be more evident in more recently discharged female patients (six to 12 months after discharge) admitted to the ICU, compared to those discharged for longer (24 months) (Hypothesis 2).

Since one-third of severe COVID-19 patients may experience long-term complications and a higher risk of premature death [36], the present study has direct clinical relevance for hospitalized COVID-19 patients. These findings may help identify the impact of psychological and cognitive factors on QoL among discharged patients dealing with trauma-related stress. Finally, findings are expected to inform the development of long-term health strategies directed to post-COVID-19 trauma, particularly by addressing cognitive impairment and QoL.

## 2. Methods

### 2.1. Conceptual Model

**Figure 1:**
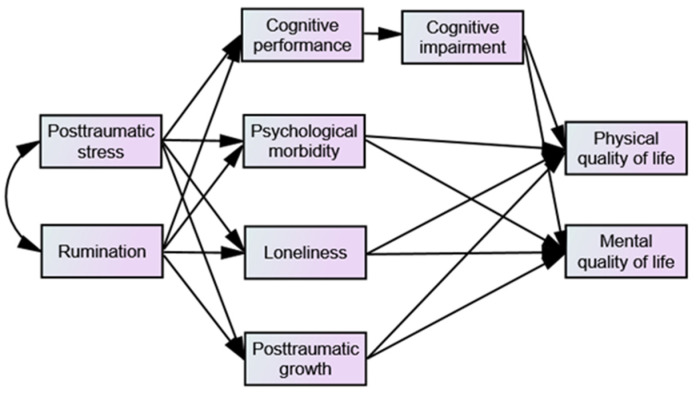
Conceptual model.

This study is based on the Cognitive Aging Model [37], which postulates that stress-related unconstructive repetitive thinking may act as a psychological mechanism that extends physiological and emotional responses to chronic stress, negatively affecting cognitive function. Focusing on patients’ QoL, the present study evaluated the mediator effect of cognitive (cognitive performance and impairment) and emotional (psychological morbidity, loneliness, and PTG) variables in the relationship between PTSS/rumination and QoL (Figure 1).

### 2.2. Study Design and Participants

This study is a prospective cohort study of patients hospitalized with COVID-19 during typical spike months: October 2020 to March 2021 (cohort I), October 2021 to March 2022 (cohort II), and October 2022 to March 2023 (cohort III). Due to a significant reduction in COVID-19 hospitalizations during cohorts II and III, these two periods were combined. As a result, this study includes two cohorts: October 2020 to March 2021 (cohort I) and October 2021 to March 2023 (cohort II). Data collection occurred in a central hospital in northern Portugal between January 2023 and March 2024.

Patients were informed by their physicians about the study’s purpose, and those who volunteered to participate were introduced to the research team. Patients were eligible for inclusion if they met the following criteria: (i) being between 18 and 75 years old, (ii) having a diagnosis of COVID-19 infection as the primary or secondary reason for hospitalization, (iii) being hospitalized during the cohort periods, and (iv) followed in the outpatient infectious diseases clinic of the hospital. Patients were not eligible if they (i) had psychiatric disorders, dementia, or brain injury (recorded in the patient’s medical records), (ii) were under chemotherapy or radiotherapy treatment, or (iii) were long-term residents at a skilled nursing facility. Participation was voluntary with no compensation.

The study protocol was approved by the Ethics Committee of the central hospital where data collection took place [Ref. 2022.069(054-DEFI/055-CE)]. All participants were informed and signed a consent form. The study was conducted according to the Declaration of Helsinki.

### 2.3. Measures

Sociodemographic and Clinical Questionnaire. This questionnaire was created for this study to gather information on sociodemographic (e.g., sex, age, marital status, education level, employment status) and clinical (e.g., hospitalization duration, time since discharge, ICU admission) characteristics of patients.

Impact of Event Scale—Revised (IES-R) [38]. IES-R is a 22-item measure to assess PTSS based on the Diagnostic and Statistical Manual of Mental Disorders criteria for PTSD. This scale includes three subscales: Intrusion (8 items; e.g., “Pictures about it popped into my mind”), Avoidance (8 items; e.g., “I tried not to think about it”), and Hyperarousal (6 items; e.g., “I was jumpy and easily startled”). Participants were asked to rate each item on a 5-point scale (from 0 = “not at all” to 4 = “extremely”), considering the past week. This study used the IES-R global score, with higher scores indicating more PTSS. Cronbach’s alpha for the Portuguese IES-R version was 0.95, whereas in the present study it was 0.97.

Event-Related Rumination Inventory (ERRI) [39]. This scale comprises 20 items that measure two styles of rumination related to PTG: Intrusive thoughts (10 items; e.g., “I thought about the event when I did not mean to”) and Deliberate rumination (10 items; e.g., “I forced myself to deal with my feelings about the event”). For each item, participants rated the degree to which the thoughts occurred during the weeks immediately after the traumatic event, using a 4-point Likert scale (from 0 = “not at all” to 3 = “often”). Higher scores indicate a higher frequency of Intrusive and Deliberate ruminations. Cronbach’s alpha for the Portuguese ERRI version was 0.94 for the total scale, 0.95 for Intrusion, and 0.90 for Deliberation. This study used the total scale with an alpha coefficient of 0.97.

Trail-Making Test (TMT) [40]. TMT is a timed neuropsychological test with two parts: part A (TMT-A) to measure attention, visual scanning, and speed of eye-hand coordination and information processing; part B (TMT-B) to assess working memory and executive functions. TMT-derived scores include difference score (B − A), ratio score (B/A), proportion score (B − A/A), sum score (A + B), and multiplication score (A × B/100)], all calculated based on participant’s sex, age, years of education, and time of completion. Due to the high number of participants that could not complete part B (*n* = 85; 33%), this study used TMT-A raw completion time (TMT-A time), with longer times suggesting worse cognitive performance, specifically regarding attention and processing speed.

Montreal Cognitive Assessment Scale (MoCA) [41]. This instrument is a 10-min neuropsychological test for the early detection of mild cognitive impairment. MoCA evaluates eight cognitive domains: Visuospatial/Executive, Naming, Memory, Attention, Language, Abstraction, Delayed recall, and Orientation. This instrument has a total score of 30, with scores ≤ 26 indicating cognitive impairment. The score is increased by one if the individual’s education years are less than twelve. This study used the scale’s total scores, with lower scores suggesting greater cognitive impairment.

Hospital Anxiety and Depression Scale (HADS) [42]. This scale comprises 14 items that assess psychological morbidity, including Depression (7 items; e.g., “I feel cheerful”) and Anxiety (7 items; e.g., “Worrying thoughts go through my mind”). Items are rated on a four-point scale, with higher scores indicating greater symptom severity. In addition to subscales scores, a global HADS score has been validated as an index of psychological morbidity or distress in several clinical populations [43,44]. Cronbach’s alphas for the Portuguese version were 0.81 for Depression and 0.76 for Anxiety. This study used the global score, presenting a Cronbach’s alpha of 0.87.

UCLA Loneliness Scale-16 (UCLA) [45]. UCLA was used to assess feelings of loneliness. This scale includes 16 items, divided into two factors: Social isolation (e.g., “I feel abandoned”) and Affinity (e.g., “I feel a lack of company”). Items are scored on a Likert scale of 1 (“never”) to 4 (“often”), with higher scores indicating greater loneliness. Cronbach’s alphas for the Portuguese UCLA were 0.93 for the overall scale, 0.92 for Social isolation, and 0.82 for Affinities. This study used the total scale, with a Cronbach’s alpha of 0.94.

Posttraumatic Growth Inventory (PTGI) [46]. PTGI contains 10 items to evaluate personal growth after adversity. Items are divided into five dimensions: Relating to others (2 items; e.g., “I have a greater sense of closeness with others”), New possibilities (2 items; “I established a new path for my life”), Personal strength (2 items; e.g., “I know better that I can handle difficulties”), Spiritual change (2 items; e.g., “I have a stronger religious faith”), and Appreciation of life (2 items; “I changed my priorities about what is important in life”). The response scale is a 6-point Likert scale that was adapted for this study, ranging from 0 (“I did not experience this change as a result of the pandemic”) to 5 (“I experienced this change to a very great degree as a result of the pandemic”). This study used the global score, with higher scores reflecting greater PTG. Internal consistency for the total score was 0.88 in the Portuguese version, and 0.93 in this study.

Short-Form Health Survey (SF-12) [47]. SF-12 assessed health-related QoL through eight health dimensions: Physical function, Physical performance, Bodily pain, General health perceptions, Vitality, Social function, Emotional performance, and Mental health. SF-12 also provides physical (PSM) and mental (MSM) summary measures, with higher scores indicating better health-related QoL. Items are answered using a Likert scale with three or five options. Internal consistency for the Portuguese version was good (PSM: α = 0.86; MSM: α = 0.87). In this study, Cronbach’s alphas for PSM and MSM were 0.82 and 0.87, respectively.

### 2.4. Data Analysis

Continuous variables were analyzed using means and standard deviations, while categorical variables were analyzed using frequencies and percentages. Independent samples *t* tests were conducted to assess differences in all variables included in the mode based on the hospitalization diagnosis, i.e., COVID-19 as primary versus secondary diagnosis. The proposed model was tested using a path analysis with bootstrapping. Goodness-of-fit indexes were calculated to evaluate the adequacy of the model, with chi-square statistic (χ^2^/df) below 2, comparative fit index (CFI) and Tucker–Lewis index (TLI) over 0.95, root mean square error approximation (RMSEA) below 0.07, and standardized root mean square residual (SRMR) below 0.08, reflecting a good fit [48]. The indirect paths were tested with 5000 bootstrap samples and a 95% confidence interval. Furthermore, multigroup analyses were conducted to test the moderating effect of sex (male versus female), time since discharge (last six and 12 months versus 24 months), and admission to the ICU (yes versus no) as dichotomous variables. All results report unstandardized regression coefficients. Finally, a *p*-value of less than 0.05 indicated statistical significance.

All statistical analyses were performed using SPSS Software (version 28.0) and SPSS AMOS (version 28.0).

### 2.5. Sample Size

A power analysis was performed with power set at 0.80, significance at 5%, a medium effect size, and seven independent variables (PTSS, rumination, psychological morbidity, loneliness, PTG, cognitive performance, and cognitive impairment), requiring a sample size of 103 participants [49].

## 3. Results

### 3.1. Sample Characteristics

Table 1 describes participants’ sociodemographic, clinical, and psychological variables. Patients (*N* = 258) were mainly men (65.9%), on average 61 years old, and professionally inactive (62.8%). Most patients were hospitalized during the first cohort (24 months before assessment) (46.6%) due to a COVID-19 diagnosis (75.2%).

Regarding patients’ hospitalization diagnosis (COVID-19 as primary versus secondary diagnosis), there were no differences in PTSS (*t* (256) = −0.758, *p* = 0.449), rumination (*t* (256) = −0.800, *p* = 0.425), cognitive performance (*t* (256) = 0.355, *p* = 0.723), cognitive impairment (*t* (256) = 0.532, *p* = 0.595), psychological morbidity (*t* (256) = 1.484, *p* = 0.139), loneliness (*t* (256) = 1.920, *p* = 0.056), PTG (*t* (256) = 0.334, *p* = 0.739), physical QoL (*t* (256) = −0.587, *p* = 0.558), and mental QoL (*t* (256) = 0.406, *p* = 0.685).

### 3.2. Path Analysis Model

**Figure 2:**
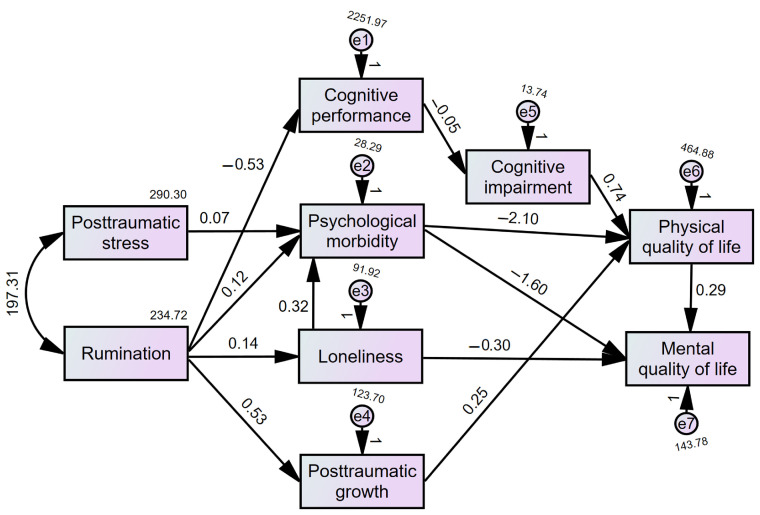
Final adjusted model.

The estimated values of the fit indices indicated that the global model fit was not adequate: χ^2^/df = 8.724, CFI = 0.852, TLI = 0.719, RMSEA = 0.173 [0.150, 0.198]; SRMR = 0.107. Subsequently, several pathways were reevaluated according to the modification indices, the significance of the path coefficients, and the final model adjustment. Specifically, non-significant paths (*p* > 0.05) were removed from the model. After their removal, two modification indices remained and were incorporated, as both suggestions were theoretically supported and improved the overall fit of the model, resulting in the addition of two pathways (loneliness → psychological morbidity; physical QoL → mental QoL) (Figure 2). The adjusted model revealed a good fit to the data: χ^2^/df = 1.584, CFI = 0.987, TLI = 0.979, RMSEA = 0.048 [0.010, 0.076]; SRMR = 0.053 (Figure 2).

All direct paths were significant at *p* < 0.05 (Table 2). From the results, the standardized effect of rumination on PTG was more than twice that of rumination on cognitive performance (TMT-A time), psychological morbidity, or loneliness. The negative association between psychological morbidity and physical and mental QoL was also more than twice that of cognitive impairment (MoCA)/PTG on physical QoL, and loneliness on mental QoL. Indirect effects in the model were all statistically significant, mainly with small or medium effect sizes (Table 3). Significant indirect effects were found for rumination on mental QoL (*β* = −0.253, 95% CI [−0.359, −0.144], *p* < 0.001) and loneliness on physical (*β* = −0.251, 95% CI [−0.319, −0.187], *p* < 0.001) and mental QoL (*β* = −0.315, 95% CI [−0.391, −0.245], *p* < 0.001).

### 3.3. The Moderating Role of Sex, Time Since Discharge, and ICU Admission

The moderating analysis regarding sex showed a significant difference between the unconstrained (adjusted) model and the fully constrained model (Δχ^2^(32) = 64.412, *p* = 0.001), suggesting that sex significantly moderated the hypothesized model. Specifically, among male patients, rumination was significantly associated with psychological morbidity (*β* = 0.13, *p* = 0.02), and loneliness was negatively related to mental QoL (*β* = −0.41, *p* < 0.001). In contrast, significant relationships emerged only for female patients between rumination and cognitive performance (TMT-A time) (*β* = −0.83, *p* = 0.014), rumination and loneliness (*β* = 0.19, *p* = 0.004), and PTSS and psychological morbidity (*β* = 0.13, *p* = 0.007).

Regarding time since discharge (24 months versus 6 to 12 months), there was also a significant difference between the unconstrained (adjusted) model and the fully constrained model (Δχ^2^(32) = 78.416, *p* < 0.001), suggesting that time since discharge significantly moderated the hypothesized model. In particular, only patients discharged for a more extended period (24 months) showed significant relationships between PTSS and psychological morbidity (*β* = 0.08, *p* = 0.033), cognitive impairment and physical QoL (*β* = 0.99, *p* = 0.012), and PTG and physical QoL (*β* = 0.29, *p* < 0.041). On the contrary, the relationships between rumination and loneliness (*β* = 0.21, *p* < 0.001), and loneliness and mental QoL (*β* = −0.54, *p* < 0.001) were significant only in more recently discharged patients (6 months).

ICU admission did not moderate the hypothesized model, as there were no significant differences between the unconstrained (adjusted) model and the fully constrained model (Δχ^2^(32) = 30.650, *p* = 0.535).

## 4. Discussion

The present study examined the contribution of PTSS and rumination to QoL in patients hospitalized with COVID-19, using mediation pathways related to cognitive and emotional functioning, loneliness, and PTG. Grounded in the Cognitive Aging Model, our results substantiate the critical role of cognitive and emotional processes for recovery and adaptability following COVID-19.

Interestingly, over one-quarter of the sample (33%) failed to complete the TMT-B, suggesting that working memory and executive control may be significantly compromised in hospitalized patients with COVID-19. Given that TMT-B requires enhanced cognitive flexibility and task-switching abilities, this finding highlights its potential as a marker of post-COVID-19 executive dysfunction. Previous studies have reported that hospitalized COVID-19 patients often experience impairments in executive functions, particularly in set-shifting and split attention, akin to those assessed by TMT-B [32,50,51]. These results underscore the need to integrate cognitive assessment and specialized neurorehabilitation, aimed explicitly at executive function, into long-term post-COVID-19 care initiatives.

As expected (Hypothesis 1), PTSS and rumination correlated with poorer physical and mental QoL, chiefly due to psychological morbidity (depression and anxiety), loneliness, cognitive deterioration, and PTG. Global rumination emerged as a key predictor, contributing to QoL through several mediators. The correlation of rumination with PTG was significantly stronger than with cognitive or emotional symptoms, suggesting that rumination may concurrently represent maladaptive processes (e.g., distress, negative affect) and constructive trauma processing (e.g., growth, meaning-making), depending on its nature and intensity [52]. Considering that the effect of rumination on PTG may range between distress and growth [52], future studies should further investigate the individual contributions of intrusive and deliberate rumination on PTG.

Among the mediators, psychological morbidity, specifically symptoms of depression and anxiety, emerged as the most significant contributor to QoL outcomes. This finding aligns with previous research revealing the widespread effects of anxiety and depression in COVID-19 survivors [16,53]. For example, Silva et al. [53] found that one year after discharge, almost 20% of critical COVID-19 survivors continued to experience depression symptoms, with repercussions in several health-related QoL areas. Similarly, Egger et al. [16] showed that anxiety, despair, and exhaustion persistently impacted QoL in patients one-year post-discharge, exhibiting minimal signs of recovery. In our model, psychological symptoms were more strongly associated with both physical and mental QoL than cognitive impairment, loneliness, or PTG, underscoring the necessity for prolonged mental health assessment and intervention in this population.

Cognitive impairment, assessed through MoCA, contributed to physical QoL but not mental QoL. This result may indicate that the broad cognitive domains assessed by MoCA, such as memory, attention, and orientation, may be more closely related to physical autonomy than emotional well-being. A study by Ariza et al. [34], involving 492 participants, including 398 post-COVID-19 individuals, found that cognitive impairment was significantly associated with reduced physical QoL and overall functioning, while emotional factors, such as anxiety and depression, strongly predicted mental QoL.

The analysis of mediators revealed that the association between loneliness and mental QoL was more significant than that of PTG and cognitive impairment, indicating that social isolation may exert a more immediate and emotionally stressful effect on daily functioning. This outcome aligns with increasing evidence showing that the social repercussions of the COVID-19 pandemic, such as physical distancing, quarantine, and decreased social support, are both pervasive and psychologically detrimental [25,26]. Moreover, loneliness has been demonstrated to endure even post-recovery from COVID-19, especially in older or hospitalized individuals [28]. These findings underscore the importance of addressing social and relational aspects of post-COVID-19 treatment in addition to medical and cognitive rehabilitation.

Interestingly, although PTG was positively correlated with QoL, the effect size was comparatively smaller than that of other mediators, including psychological morbidity (depression and anxiety) and loneliness. Nevertheless, PTG emerged as a significant protective factor, particularly among patients discharged for extended periods (24 months), suggesting that the psychological benefits of personal growth may require time to manifest after trauma [23]. The temporal aspect of PTG aligns with the existing literature, indicating that such growth is more evident during longer recovery phases, as individuals are given the time and space to process their experiences and redefine the meaning of their lives. In a comprehensive review and meta-analysis, Wu et al. [24] found that moderate-to-high levels of PTG were more commonly observed in long-term follow-ups compared to acute periods. These findings underscore PTG’s role not as an immediate protective factor, but as a delayed, adaptive response to adversity. The capacity for growth may be particularly relevant in designing long-term psychosocial interventions aimed at helping survivors reinterpret their COVID-19 experiences to foster renewed purpose, strengthened connections, and a deeper appreciation of life.

Hypothesis 2 was partially confirmed since only sex and time since discharge played a moderating role. Only in female patients, rumination showed a significant correlation with cognitive performance (attention and processing speed) and loneliness, as well as between PTSS and psychological distress (depression and anxiety). These findings corroborate prior studies indicating that women may be more susceptible to the psychosocial and cognitive consequences associated with COVID-19 [9,11,12,31,54,55]. For instance, a global survey across 59 countries revealed that women reported higher levels of trauma-related distress, depression, anxiety, and stress compared to men during the pandemic [54]. Previous studies have also shown women reporting higher rates of cognitive symptoms, such as brain fog, fatigue, and memory difficulties, following COVID-19 [55]. Conversely, only male patients showed significant associations between rumination and psychological morbidity (depression and anxiety), as well as between loneliness and mental QoL. Although direct evidence regarding the specific associations in male COVID-19 patients is limited, the present findings may reflect well-documented sex disparities in emotional expressiveness and the propensity to seek assistance [56,57]. Men are typically less likely to pursue psychological support and more inclined to suppress emotional turmoil, which may increase their vulnerability to psychological strain and perceived loneliness [57]. These patterns underscore the need for sex-sensitive mental health interventions that consider differential coping mechanisms and social expectations following hospitalization for COVID-19.

Differences based on time since discharge revealed that, at 24 months post-discharge, patients showed significant associations between PTSS and psychological morbidity (depression and anxiety), and between cognitive impairment (MoCA)/PTG and physical QoL. Although we expected key pathways, such as the association between PTSS and psychological morbidity, to be stronger in more recently discharged patients, previous studies have also found higher levels of PTSS, depression, and anxiety in COVID-19 survivors two years after discharge [58,59]. This result is probably due to hospitalizations during the early phase of the pandemic that occurred under particularly stressful conditions, including higher mortality rates, limited healthcare resources, and the absence of vaccines [3]. According to previous research, cognitive problems may also last long after the acute infection phase (e.g., [59,60]). For instance, a recent longitudinal study by Taquet et al. [60] found that discharged COVID-19 patients continued to exhibit significant cognitive abnormalities and psychiatric symptoms two years after discharge, with symptoms worsening over time. Consistent with previous findings showing higher levels of rumination, increased loneliness, and reduced well-being in recently discharged COVID-19 patients (e.g., [61]), the current study found that only patients within the 6–12 months post-discharge window showed significant associations between rumination, loneliness, and reduced mental QoL. These results underscore the early post-discharge period as a critical window for early psychological and social interventions aimed at improving long-term mental health outcomes.

ICU admission did not moderate any relationships in the model, contrary to expectations, as ICU survivors are generally at increased risk for psychological symptoms [13,14] and cognitive impairment [62]. However, previous studies have shown that both ICU and non-ICU COVID-19 patients may experience similarly significant psychological and cognitive difficulties [63], possibly due to the pandemic-related hospital measures such as prolonged isolation, uncertainty, and limited access to support. Further research is needed to confirm these findings.

### Limitations and Future Studies

While the findings of this study shed light on the psychological and cognitive pathways associated with post-COVID-19 QoL, some limitations should be acknowledged. The sample was recruited from a single hospital in Portugal, which may limit the generalizability of the findings, despite the hospital’s central role in the region’s healthcare system. Although the study employed a prospective design, all assessments (except cognitive tests) were self-reported, which may have introduced response bias or reflected subjective interpretation rather than objective symptoms. While rumination and PTSS were assessed on QoL outcomes, causal inferences are constrained due to the correlational nature of the mediation model since directional or causal relationships between variables may not be established. The non-inclusion of TMT-B performance scores did not allow us to assess set-shifting, a key cognitive domain often affected in post-COVID-19 conditions. While the 6- to 24-month timeframe provides insight into the healing phases, more extended follow-up periods are required to understand the long-term trajectories of cognitive decline, psychological distress, loneliness, and PTG following COVID-19. Finally, to decrease type I error, the path model was based on a theoretical model, and bootstrapping was used to provide more accurate confidence intervals and *p*-values for indirect effects. Nonetheless, findings should be interpreted with caution.

Future studies should employ longitudinal designs with larger, multi-site samples, and include biomarkers or neuroimaging, to validate cognitive and affective findings, and investigate intervention-based techniques to reduce the impact of PTSS and rumination. Individual variables, such as patients’ socioeconomic level, clinical and cultural characteristics, and pre-existing mental health disorders, should also be considered when assessing post-COVID-19 recovery.

## 5. Conclusions and Implications

This study showed that PTSS and rumination negatively contributed to the QoL of hospitalized patients with COVID-19 through impaired cognitive functioning, psychological distress (depression and anxiety), loneliness, and PTG, with sex and time since discharge showing moderating effects. These findings underscore the need to incorporate mental health screening, cognitive rehabilitation, and psychological support into follow-up care, particularly for high-risk groups, as well as the importance of tailored post-COVID-19 interventions according to sex and time since discharge. For those survivors with high social isolation conditions, occupational therapy interventions addressing loneliness should be made accessible to help reduce any impacts on QoL. Including occupational therapy in multidisciplinary rehabilitation teams, even with teleoccupational therapy, showed promising results on cognitive function and QoL [64].

## Figures and Tables

**Table 1:** Sample characteristics (*N* = 258).

Sociodemographic Variables		Patients
		***n* (%)/M ± SD**
Sex	Women	88 (34.10)
	Men	170 (65.90)
Age		61.41 ± 10.86
	≤49	32 (12.40)
	50–59	59 (22.90)
	60–69	98 (38.00)
	70–75	69 (26.70)
Marital status	Married/non-marital partnership	162 (62.80)
	Single/divorced/widowed	96 (37.20)
Education	≤Primary education	101 (39.20)
	≤Secondary education	121 (46.90)
	≤University degree	36 (13.90)
Professional situation	Employed	96 (37.20)
	Unemployed	24 (9.30)
	Retired	138 (53.50)
**Clinical Variables**		
Hospitalization main diagnosis	COVID-19	194 (75.20)
	Other	64 (24.80)
Intensive care unit admission	Yes	60 (23.30)
	No	198 (76.70)
Cohort	I	120 (46.50)
	II	138 (53.50)
Discharge duration (days)		629.27 (259.33)
**Psychological Variables**	**Min.–Max.**	**M ± SD**
Posttraumatic stress (IES-R)	0.00–82.00	13.90 (17.07)
Rumination (ERRI)	0.00–60.00	16.54 (15.35)
Cognitive performance (TMT-A time)	26.00–375.00	83.81 (48.25)
Cognitive impairment (MoCA)	8.00–29.00	20.24 (4.43)
Psychological morbidity (HADS)	0.00–34.00	9.38 (7.10)
Loneliness (UCLA)	16.00–63.00	24.89 (9.83)
Posttraumatic growth (PTGI)	0.00–50.00	17.95 (13.79)
Physical QoL (SF-12)	0.00–100.00	58.01 (26.20)
Mental QoL (SF-12)	6.25–100.00	68.62 (21.85)

**Table 2:** Standardized direct effects in the final adjusted model.

Path	b (SE)	Z Value	*p*	*β*
Posttraumatic stress (IES-R) → Psych. morbidity (HADS)	0.074 (0.030)	2.490	0.013	0.178
Rumination (ERRI) → Cognitive performance (TMT-A)	−0.535 (0.193)	−2.767	0.006	−0.170
Rumination (ERRI) → Psych. morbidity (HADS)	0.122 (0.033)	3.651	<0.001	0.263
Rumination (ERRI) → Loneliness (UCLA)	0.135 (0.039)	3.464	<0.001	0.211
Rumination (ERRI) → Posttraumatic growth (PTGI)	0.528 (0.045)	11.658	<0.001	0.588
Loneliness (UCLA) → Psych. morbidity (HADS)	0.318 (0.035)	9.180	<0.001	0.438
Cognitive performance (TMT-A) → Cognitive impairment (MoCA)	−0.050 (0.005)	−10.397	<0.001	−0.544
Cognitive impairment (MoCA) → Physical QoL (SF-12)	0.737 (0.305)	2.416	0.016	0.125
Psych. morbidity (HADS) → Physical QoL (SF-12)	−2.095 (0.198)	10.580	<0.001	−0.572
Psych. morbidity (HADS) → Mental QoL (SF-12)	−1.596 (0.140)	−11.418	<0.001	−0.519
Loneliness (UCLA) → Mental QoL (SF-12)	−0.300 (0.089)	−3.353	<0.001	−0.135
Posttraumatic growth (PTGI) → Physical QoL (SF-12)	0.248 (0.102)	2.430	0.015	0.131
Physical QoL (SF-12) → Mental QoL (SF-12)	0.293 (0.034)	8.635	<0.001	0.348

Note. b = unstandardized path coefficient; SE = standard error; *β* = standardized path coefficient.

**Table 3:** Standardized indirect effects in the final adjusted model.

Predictor	Indirect Effect	Outcome	*β*	CI_95%_
Posttraumatic stress (PTSS)	Psych. morbidity (HADS)	Physical QoL (SF-12)	−0.102 *	[−0.216, −0.003]
Posttraumatic stress (PTSS)	Psych. morbidity (HADS)	Mental QoL (SF-12)	−0.128 *	[−0.273, −0.002]
Rumination (ERRI)	Loneliness (UCLA)	Psych. morbidity (HADS)	0.093 ***	[0.040, 0.159]
Rumination (ERRI)	Cognitive performance (TMT-A time)	Cognitive impairment (MoCA)	0.093 ***	[0.046, 0.144]
Rumination (ERRI)	Cognitive performance (TMT-A time) → Cognitive impairment (MoCA) [Loneliness (UCLA)→] Psych. morbidity (HADS)	Physical QoL (SF-12)	−0.115 *	[−0.225, −0.012]
Rumination (ERRI)	Cognitive performance (TMT-A time) → Cognitive impairment (MoCA) → Physical QoL (SF-12) [Loneliness (UCLA) →] Psych. morbidity → Physical QoL (SF-12)	Mental QoL (SF-12)	−0.253 ***	[−0.359, −0.144]
Loneliness (UCLA)	Psych. morbidity (HADS)	Physical QoL (SF-12)	−0.251 ***	[−0.319, −0.187]
Loneliness (UCLA)	Psych. morbidity (HADS)	Mental QoL (SF-12)	−0.315 ***	[−0.391, −0.245]
Psych. morbidity (HADS)	Physical QoL (SF-12)	Mental QoL (SF-12)	−0.199 ***	[−0.262, −0.147]
Cognitive performance (TMT-A time)	Cognitive impairment (MoCA)	Physical QoL (SF-12)	−0.068 *	[−0.128, −0.010]
Cognitive performance (TMT-A time)	Cognitive impairment (MoCA)	Mental QoL (SF-12)	−0.024 *	[−0.045, −0.004]
Cognitive impairment (MoCA)	Physical QoL (SF-12)	Mental QoL (SF-12)	0.044 *	[0.007, 0.081]
Posttraumatic growth (PTGI)	Physical QoL (SF-12)	Mental QoL (SF-12)	0.046 *	[0.008, 0.087]

Note. * *p* < 0.05; *** *p* < 0.001; *β* = standardized path coefficient; CI_95%_ = bootstrap bias-corrected confidence interval at 95% (5000 samples), lower and upper.

## Data Availability

The data are available and can be provided upon request.

## References

[1] Mallah S.I., Ghorab O.K., Al-Salmi S., Abdellatif O.S., Tharmaratnam T., Iskandar M.A., Sefen J.A.N., Sidhu P., Atallah B., El-Lababidi R. (2021). COVID-19: Breaking down a global health crisis. Ann. Clin. Microbiol. Antimicrob..

[2] Worldometer COVID-19 Coronavirus Pandemic: Portugal. https://www.worldometers.info/coronavirus/country/portugal/.

[3] Ferreira D.C., Marques R.C., Nunes A.M. (2023). The Portuguese Public Hospitals Performance Evolution before and during the SARS-CoV-2 Pandemic (2017–2022). Sustainability.

[4] Medical Xpress Portugal’s COVID-19 Crisis Slams Hospitals. 3 February 2021. https://medicalxpress.com/news/2021-02-portugal-covid-slams-hospitals.html.

[5] Horesh D., Brown A.D. (2020). Traumatic stress in the age of COVID-19: A call to close critical gaps and adapt to new realities. Psychol. Trauma.

[6] Kaubisch L.T., Reck C., von Tettenborn A., Woll C.F.J. (2022). The COVID-19 pandemic as a traumatic event and the associated psychological impact on families—A systematic review. J. Affect. Disord..

[7] Bridgland V.M.E., Moeck E.K., Green D.M., Swain T.L., Nayda D.M., Matson L.A., Hutchison N.P., Takarangi M.K.T. (2021). Why the COVID-19 pandemic is a traumatic stressor. PLoS ONE.

[8] Kira I.A., Shuwiekh H.A.M., Ashby J.S., Elwakeel S.A., Alhuwailah A., Sous M.S.F., Baali S.B.A., Azdaou C., Oliemat E.M., Jamil H.J. (2023). The Impact of COVID-19 Traumatic Stressors on Mental Health: Is COVID-19 a New Trauma Type. Int. J. Ment. Health Addict..

[9] Husky M.M., Pietrzak R.H., Marx B.P., Mazure C.M. (2021). Research on Posttraumatic Stress Disorder in the Context of the COVID-19 Pandemic: A Review of Methods and Implications in General Population Samples. Chronic Stress.

[10] COVID-19 Mental Disorders Collaborators (2021). Global prevalence and burden of depressive and anxiety disorders in 204 countries and territories in 2020 due to the COVID-19 pandemic. Lancet.

[11] Chen Y., Huang X., Zhang C., An Y., Liang Y., Yang Y., Liu Z. (2021). Prevalence and predictors of posttraumatic stress disorder, depression, and anxiety among hospitalized patients with coronavirus disease 2019 in China. BMC Psychiatry.

[12] Lovik A., González-Hijón J., Kähler A.K., Valdimarsdóttir U.A., Frans E.M., Magnusson P.K.E., Pedersen N.L., Hall P., Czene K., Sullivan P.F. (2023). Mental health indicators in Sweden over a 12-month period during the COVID-19 pandemic—Baseline data of the Omtanke2020 Study. J. Affect. Disord..

[13] Hussain N., Samuelsson C.M., Drummond A., Persson C.U. (2024). Prevalence of symptoms of anxiety and depression one year after intensive care unit admission for COVID-19. BMC Psychiatry.

[14] Nagarajan R., Krishnamoorthy Y., Basavarachar V., Dakshinamoorthy R. (2022). Prevalence of post-traumatic stress disorder among survivors of severe COVID-19 infections: A systematic review and meta-analysis. J. Affect. Disord..

[15] Becerra-Canales B., Campos-Martínez H.M., Campos-Sobrino M., Aquije-Cárdenas G.A. (2022). Trastorno de estrés postraumático y calidad de vida del paciente post-COVID-19 en Atención Primaria [Post-traumatic stress and QoL of post-COVID-19 patients in primary care]. Aten. Primaria.

[16] Egger M., Wimmer C., Stummer S., Reitelbach J., Bergmann J., Müller F., Jahn K. (2024). Reduced health-related QoL, fatigue, anxiety and depression affect COVID-19 patients in the long-term after chronic critical illness. Sci. Rep..

[17] Juczyński Z., Kwiecińska L., Ogińska-Bulik J. (2023). Ruminations as predictors of post-traumatic stress disorder after hospitalization for COVID-19. Ruminacje jako wyznaczniki zespołu stresu pourazowego po hospitalizacji z powodu COVID-19. Psychiatr. Pol..

[18] Satici B., Saricali M., Satici S.A., Griffiths M.D. (2022). Intolerance of Uncertainty and Mental Wellbeing: Serial Mediation by Rumination and Fear of COVID-19. Int. J. Ment. Health Addict..

[19] Kang H.S., Kim B.N. (2021). The Role of Event-Related Rumination and Perceived Social Support on Psychological Distress during the COVID-19 Pandemic: Results from Greater Daegu Region in South Korea. Psychiatry Investig..

[20] Tedeschi R.G., Shakespeare-Finch J., Taku K., Calhoun L.G. (2018). Posttraumatic Growth: Theory, Research, and Applications.

[21] Bovero A., Balzani S., Tormen G., Malandrone F., Carletto S. (2023). Factors Associated with Post-Traumatic Growth during the COVID-19 Pandemic: A Systematic Review. J. Clin. Med..

[22] Adjorlolo S., Adjorlolo P., Andoh-Arthur J., Ahiable E.K., Kretchy I.A., Osafo J. (2022). Post-Traumatic Growth and Resilience among Hospitalized COVID-19 Survivors: A Gendered Analysis. Int. J. Environ. Res. Public Health.

[23] Pietrzak R.H., Tsai J., Southwick S.M. (2021). Association of Symptoms of Posttraumatic Stress Disorder with Posttraumatic Psychological Growth Among US Veterans During the COVID-19 Pandemic. JAMA Netw. Open.

[24] Wu X., Kaminga A.C., Dai W., Deng J., Wang Z., Pan X., Liu A. (2019). The prevalence of moderate-to-high posttraumatic growth: A systematic review and meta-analysis. J. Affect. Disord..

[25] Ernst M., Niederer D., Werner A.M., Czaja S.J., Mikton C., Ong A.D., Rosen T., Brähler E., Beutel M.E. (2022). Loneliness before and during the COVID-19 pandemic: A systematic review with meta-analysis. Am. Psychol..

[26] Sankaran S., Sankaran S. (2023). The Loneliness Epidemic and How It Affects Hospitalized Patients. Health Equity in Hospital Medicine.

[27] Madden R., Ahmed R., Cloonan J., May E., Chambers H., Briggs R. (2022). 347 Loneliness Amonst Older Inpatients in the Context of COVID-Related Visiting Restrictions. Age Ageing.

[28] Sipowicz K., Pietras T., Mosiołek A., Sobstyl M., Ring M., Kamecki K., Stefańczyk I., Kosmalski M. (2023). The sense of loneliness and meaning in life in post-COVID convalescents—A preliminary study. Front. Psychiatry.

[29] Hawkley L.C. (2022). Loneliness and health. Nat. Rev. Dis. Primers.

[30] Crivelli L., Palmer K., Calandri I., Guekht A., Beghi E., Carroll W., Frontera J., García-Azorín D., Westenberg E., Winkler A.S. (2022). Changes in cognitive functioning after COVID-19: A systematic review and meta-analysis. Alzheimer’s Dement..

[31] Vasilevskaya A., Mushtaque A., Tsang M.Y., Alwazan B., Herridge M., Cheung A.M., Tartaglia M.C. (2023). Sex and age affect acute and persisting COVID-19 illness. Sci. Rep..

[32] Jaywant A., Vanderlind W.M., Alexopoulos G.S., Fridman C.B., Perlis R.H., Gunning F.M. (2021). Frequency and profile of objective cognitive deficits in hospitalized patients recovering from COVID-19. Neuropsychopharmacology.

[33] Wood G.K., Sargent B.F., Ahmad Z.U., Tharmaratnam K., Dunai C., Egbe F.N., Martin N.H., Facer B., Pendered S.L., Rogers H.C. (2025). Posthospitalization COVID-19 cognitive deficits at 1 year are global and associated with elevated brain injury markers and gray matter volume reduction. Nat. Med..

[34] Ariza M., Cano N., Segura B., Bejar J., Barrué C., Cortés C.U., Junqué C., Garolera M., NAUTILUS Project Collaborative Group (2024). Cognitive and emotional predictors of QoL and functioning after COVID-19. Ann. Clin. Transl. Neurol..

[35] Szewczyk W., Fitzpatrick A.L., Fossou H., Gentile N.L., Sotoodehnia N., Vora S.B., West T.E., Bertolli J., Cope J.R., Lin J.S. (2024). Long COVID and recovery from Long COVID: QoL impairments and subjective cognitive decline at a median of 2 years after initial infection. BMC Infect. Dis..

[36] O’Dowd A. (2021). COVID-19: Third of people infected have long term symptoms. BMJ.

[37] Scott S.B., Graham-Engeland J.E., Engeland C.G., Smyth J.M., Almeida D.M., Katz M.J., Lipton R.B., Mogle J.A., Munoz E., Ram N. (2015). The Effects of Stress on Cognitive Aging, Physiology and Emotion (ESCAPE) Project. BMC Psychiatry.

[38] Lopes A., Rocha J. (2013). Convergent Validity of Impact of Event Scale-Revised and Impact of Event Scale-6 Portuguese Versions. Master’s Thesis.

[39] Ramos C., Figueiras L., Lopes M., Leal I., Tedeschi R. (2015). Inventário de ruminação relacionada com o acontecimento: Qualidades psicométricas na população portuguesa [Event-Related Rumination Inventory: Psychometric Properties on a Portuguese Sample]. Psicol. Saúde Doenças.

[40] Cavaco S., Gonçalves A., Pinto C., Almeida E., Gomes F., Moreira I., Fernandes J., Teixeira-Pinto A. (2013). Trail Making Test: Regression-based norms for the portuguese population. Arch. Clin. Neuropsychol..

[41] Freitas S., Simões M.R., Martins C., Vilar M., Santana I. (2010). Estudos de adaptação do Montreal Cognitive Assessment (MoCA) para a população portuguesa [Adaptation studies of the Montreal Cognitive Assessment (MoCA) to the portuguese population]. Aval. Psicol..

[42] Pais-Ribeiro J., Silva I., Ferreira T., Martins A., Meneses R., Baltar M. (2007). Validation study of a Portuguese version of the Hospital Anxiety and Depression Scale. Psychol. Health Med..

[43] Carmichael J., Spitz G., Gould K.R., Johnston L., Samiotis A., Ponsford J. (2023). Bifactor analysis of the Hospital Anxiety and Depression Scale (HADS) in individuals with traumatic brain injury. Sci. Rep..

[44] Gibbons C.J., Mills R.J., Thornton E.W., Ealing J., Mitchell J.D., Shaw P.J., Talbot K., Tennant A., Young C.A. (2021). Rasch analysis of the hospital anxiety and depression scale (HADS) for use in motor neurone disease. Health Qual. Life Outcomes.

[45] Faustino B., Lopes P., Oliveira J., Campaioli G., Rondinone M., Bomfim H., Germano L. (2019). Psychometric and Rash Analysis of the UCLA Loneliness Scale-16 in a Portuguese Sample of Older Adults. Psychol. Stud..

[46] Lamela D., Figueiredo B., Bastos A., Martins H. (2013). Posttraumatic Growth Inventory Short Form-Portuguese Version (Portuguese PTGI-SF, PTGI-SF).

[47] Ferreira P.L., Ferreira L.N., Pereira L.N. (2012). Medidas sumário física e mental de estado de saúde para a população portuguesa [Physical and mental summary measures of health state for the portuguese population]. Rev. Port. Saúde Pública.

[48] Hair J.F., Black W., Babin B., Anderson R. (2020). Multivariate Data Analysis: A Global Perspective.

[49] Soper D.S. (2019). A-priori Sample Size Calculator for Hierarchical Multiple Regression [Software]. http://www.danielsoper.com/statcalc.

[50] Bertuccelli M., Ciringione L., Rubega M., Bisiacchi P., Masiero S., Del Felice A. (2022). Cognitive impairment in people with previous COVID-19 infection: A scoping review. Cortex.

[51] Panagea E., Messinis L., Petri M.C., Liampas I., Anyfantis E., Nasios G., Patrikelis P., Kosmidis M. (2025). Neurocognitive Impairment in Long COVID: A Systematic Review. Arch. Clin. Neuropsychol..

[52] Mertens L., Tamm G., Hoorelbeke K. (2025). Ruminative thinking styles differentially relate to posttraumatic stress versus growth following trauma exposure. Psychol. Trauma.

[53] Silva J., Martins S., Ferreira A.R., Fernandes J., Vieira T., Fontes L., Reis N., Braga A., Coimbra I., Paiva J.A. (2022). Depression and health-related quality of life in critical COVID-19 survivors. Eur. Psychiatry.

[54] Kolakowsky-Hayner S.A., Goldin Y., Kingsley K., Alzueta E., Arango-Lasprilla J.C., Perrin P.B., Baker F.C., Ramos-Usuga D., Constantinidou F. (2021). Psychosocial Impacts of the COVID-19 Quarantine: A Study of Gender Differences in 59 Countries. Medicina.

[55] Gorenshtein A., Leibovitch L., Liba T., Stern S., Stern Y. (2024). Gender Disparities in Neurological Symptoms of Long COVID: A Systematic Review and Meta-Analysis. Neuroepidemiology.

[56] Wollast R., Lüders A., Nugier A., Guimond S., Phillips J.B., Sutton R.M., Douglas K.M., Sengupta N.K., Lemay E.P., Zand S. (2025). Gender inequality and cultural values in explaining gender differences in positive and negative emotions: A comparison of 24 countries during the COVID-19 pandemic. Curr. Psychol..

[57] Gough B., Novikova I. (2020). Mental Health, Men and Culture: How Do Sociocultural Constructions of Masculinities Relate to Men’s Mental Health Help-Seeking Behaviour in the WHO European Region?.

[58] Bi Y., Xiao Y., Pan X., Zhang Y., Yang Q., Hu L. (2023). Long-term post-traumatic stress symptoms in COVID-19 survivors and its risk factors: A two-year longitudinal cohort study. Psychiatry Res..

[59] Taquet M., Skorniewska Z., De Deyn T., Hampshire A., Trender W.R., Hellyer P.J., Chalmers J.D., Ho L.P., Horsley A., Marks M. (2024). Cognitive and psychiatric symptom trajectories 2-3 years after hospital admission for COVID-19: A longitudinal, prospective cohort study in the UK. Lancet Psychiatry.

[60] Cheetham N.J., Penfold R., Giunchiglia V., Bowyer V., Sudre C.H., Canas L.S., Deng J., Murray B., Kerfoot E., Antonelli M. (2023). The effects of COVID-19 on cognitive performance in a community-based cohort: A COVID symptom study biobank prospective cohort study. eClinicalMedicine.

[61] O’Connor D.B., Wilding S., Ferguson E., Cleare S., Wetherall K., McClelland H., Melson A.J., Niedzwiedz C., O’Carroll R.E., Platt S. (2023). Effects of COVID-19-related worry and rumination on mental health and loneliness during the pandemic: Longitudinal analyses of adults in the UK COVID-19 mental health & wellbeing study. J. Ment. Health.

[62] Ollila H., Pihlaja R., Koskinen S., Tuulio-Henriksson A., Salmela V., Tiainen M., Hokkanen L., Hästbacka J. (2022). Long-term cognitive functioning is impaired in ICU-treated COVID-19 patients: A comprehensive controlled neuropsychological study. Crit. Care.

[63] Pihlaja R.E., Kauhanen L.S., Ollila H.S., Tuulio-Henriksson A.S., Koskinen S.K., Tiainen M., Salmela V.R., Hästbacka J., Hokkanen L.S. (2023). Associations of subjective and objective cognitive functioning after COVID-19: A six-month follow-up of ICU, ward, and home-isolated patients. Brain Behav. Immun. Health.

[64] Schröder D., Stölting A., Müllenmeister C., Behrens G.M.N., Klawitter S., Klawonn F., Cook A., Wegner N., Wetzke M., Schmachtenberg T. (2025). Improvement in quality of life and cognitive function in Post-COVID syndrome after online occupational therapy: Results from a randomized controlled pilot study. PLoS ONE.

